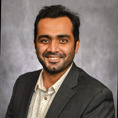# Introducing the Rising Stars of GeoHealth

**DOI:** 10.1029/2024GH001053

**Published:** 2024-04-26

**Authors:** Thanh H. Nguyen

**Affiliations:** ^1^ Editor‐in‐Chief of GeoHealth Ivan Racheff Endowed Professor of Civil and Environmental Engineering University of Illinois at Urbana‐Champaign Urbana IL USA

## Abstract

Early career researchers often asked me: how I became the editor‐in‐chief, what editors, associate editors, and editors do, why I wanted to become an editor, how much time an editor committed, would I rather spend more time on my research and publish another paper or my personal life? All of these questions make sense. When I started as an assistant professor nearly 20 years ago, I did not plan to become an editor; I wanted to do my research and teach to achieve tenure. Sound familiar? Fortunately, I was gradually pulled into the publishing process not as an author but as a reviewer, associate editor, and eventually editor‐in‐chief by several senior colleagues, for whom I am forever grateful. Now, it is my turn to prepare the next generation of editors, the backbone of science.

When I applied to be the editor‐in‐chief of GeoHealth, I proposed that I would like to establish an early career editorial board (ECEB) whose members are the rising stars of our field. Once I was selected and prepared to start my term, I started working on a proposal to the AGU publication to establish an editorial career editorial board. The main goal of this board is training to become editors. While finding reviewers and making decisions based on the reviewers' recommendations are the main duties of the editors, there are also other tasks related to publication. For example, the journal's visibility influences potential reviewers' decisions. Qualified reviewers will not likely spend time reviewing for an obscure journal publishing articles without potential impacts or values. The quality of the published articles is the most important factor in the journal's visibility. Social and digital media help the articles to reach a much wider audience outside our own circle of scientists. Ultimately, whatever we do, our word serves humanity. For this reason, our work needs to be known by non‐scientists. I am working with AGU to find a way to train the ECEB in science communication. The skills in science communication will serve you well in your research. The days that scientists only talk to each other are over. We need to learn how to communicate with policymakers and the general public. Who will fund our research? Certainly not other scientists!

The GeoHealth editorial board and I will start training the first cohort of ECEB. We will show you how to find reviewers, analyze their comments, and work with authors to get the best papers for publication. After this training, we hope to gradually move you into the role of editor. Current editors will step down from the board and move into the next chapter of their careers. You will be the ones who replace us, and the future is in your hands.

I plan to work with ECEB to develop the next chapter of GeoHealth. We received an excellence inheritance from the GeoHealth pioneers, including Dr. Rital Colwell, the founding editor, and Dr. Gabrial Phillipe, the inaugural editor‐in‐chief. Here is some food for thought. We have learned so much over the years about the problems of earth processes, including the effects of extreme weather events due to climate change on human health. Should we spend more time now finding science‐based, human‐centered solutions to these problems? What should we do to come up with implementable solutions? Our colleagues at CDC have a OneHealth framework based mainly on disease surveillance in humans, animals, and the environment. Our solutions to prevent diseases must include an understanding of earth processes, which influence the health and well‐being of all living creatures, including humans. Who knows this knowledge better than GeoHealth researchers? I propose a new framework that I hope to develop with the ECEB. It is called OneEarth OneHealth.

I am committed to having an inclusive and diverse editorial board because that is the nature of our field. We work at the intersection of geoscience and public health. Nearly all of our work is relevant to protecting human health. Our contribution is as important as that of medical doctors who treat diseases. We prevent disease from happening or at least reduce the severity of diseases. For the first cohort of ECEB, which will have an appointment for 3 years, I have recruited and interviewed the rising stars of GeoHealth with different expertise, backgrounds, and from different countries. The first cohort's membership depends on the proportion of countries where GeoHealth countries and reviewers come from. I plan to expand the ECEB and further increase the inclusion and diversity of the board. The next cohort will start in 2025, with more representatives from other countries besides North America. If you read this article, do research in GeoHealth, and are interested in this initiative, no matter where you are, send your CV to me, and we will discuss your potential involvement with ECEB.

**Mohan AMARASIRI**, Tohoku University, Japan. Dr. Mohan Amarasiri is an associate professor in the Department of Civil and Environmental Engineering at Tohoku University, Japan. His main research interest is water and human health. His research focuses on identification and quantification of pathogenic microorganisms in aquatic environments, estimating the human health risks of exposing to these microorganisms, and developing low‐cost infrastructure to eliminate those microorganisms from the environment.
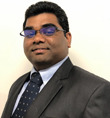


**Srinidhi Balasubramanian**, Indian Institute of Technology Bombay, India. Dr. Balasubramanian is an Assistant Professor in the Environmental Science and Engineering Department at the Indian Institute of Technology Bombay. Her research focuses on developing tools and methods to forecast air pollution at high‐spatial resolution in ambient and indoor environments. She also works at the nexus of agriculture, air quality and climate change with the aim of helping create greener and healthier food systems.
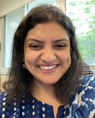


**Kyle D. Brumfield**, University of Maryland Institute for Advanced Computer Studies (UMIACS), University of Maryland, College Park, USA. Dr. Brumfield is an ORISE Postdoctoral Fellow at the University of Maryland Institute for Advanced Computer Studies (UMIACS). He has more than a decade of experience in microbial ecology, molecular genetics, and global infectious diseases related to water and health. His work helped elucidate environmental parameters that influence the occurrence and transmission of pathogenic agents related to climate change.
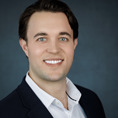


**Fang Fang**, University of Illinois, USA, Department of Urban and Regional Planning. Dr. Fang is the teaching assistant professor at University of Illinois at Urbana Champaign. Her research work primarily utilizes GIS, remote sensing, and diverse quantitative data analytic techniques, including machine learning, to comprehend urban landscape dynamics, human‐environment interactions and health under the impact of climate change. As a planner, we aim to develop a more sustainable, resilient, and healthy living environment.
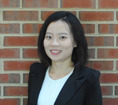


**Yun Hang**, University of Texas Health Science Center at Houston, USA. Dr. Hang is an Assistant Professor in Environmental and Occupational Health Sciences at the University of Texas Health Science Center at Houston. Over the past decade, she has worked closely with NASA science teams on the application of remote sensing in climate change, air pollution, and environmental justice research.
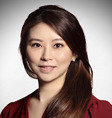


**Muhammad Maqsud Hossain**, North South University, Dhaka Bangladesh. Dr. Hossain is the Director of NSU Genome Research Institute and an Associate Professor in Bioinformatics. His leading project focuses on fighting infection and AMR leveraging artificial intelligence (AI), omics technologies, and smart sensing for diagnosis, forecasting treatment selection, and gut microbiome improvement.
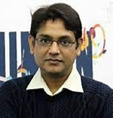


**Yaoxian Huang**, Wayne State University, USA. Dr. Huang is an atmospheric chemist at Wayne State University. His group research focuses on the nexus of air quality‐climate change‐human health, by employing observations and state‐of‐the‐art regional and global chemical transport models and chemistry‐climate models.
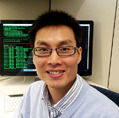


**Phong Le**, Oak Ridge National Laboratory, USA. Dr. Le is a research scientist at Oak Ridge National Laboratory and a hydroclimatologist who addresses water, climate, and human health issues. His research aims to understand the nonlinear dynamics within hydrologic and climate systems, investigating how these complex interactions at various scales impact water resources and human health, with an emphasis on infectious diseases.
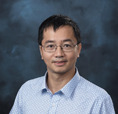


**Fangchao Liang**, Southern University of Science and Technology, China. Dr. Liang's is an Assistant Professor in the School of Public Health and Emergency Management & School of Environmental Science and Engineering (joint appointment). Her research focuses on applying high resolution satellite aerosol remote sensing data in monitoring and predicting ground level air pollutants and assessing associations between environmental exposure and health risks.
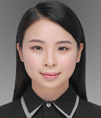


**Jennifer Stowell**, Center for Climate and Health, Boston University, USA. Dr. Stowell is a research scientist in the Center for Climate and Health at the Boston University School of Public Health. She has more than a decade of experience engaging in public health research and exposures related to climate change. Her research primarily focuses on the health impacts of wildfire smoke exposure and aims to inform disaster planning and policy measures to reduce the health risk from exposure to smoke‐specific pollutants.
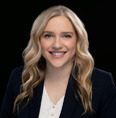


**Christopher Tessum**, University of Illinois, USA. Dr. Tessum is an Assistant Professor in the Department of Civil and Environmental Engineering at UIUC. His research focuses on modeling air pollution and its health impacts, quantifying inequities in the distribution of those impacts, and proposing and testing solutions. He studies the relationships between emissions, the human activities that cause them, and the resulting health impacts, and he develops modeling capabilities to enable these types of analyses.
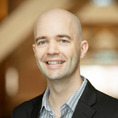


**Moiz Usmani**, University of Florida USA. Dr. Usmani is a Postdoctoral Research Associate at the University of Florida. With 10 years of experience in hydro‐epidemiology, he has actively engaged in research at the intersection of climate variability, epidemiology, mathematics, and remote sensing. His research aims to drive positive societal change by advancing the field of health ecology.